# Correction: Longitudinal diffusion barriers imposed by myofilaments and mitochondria in murine cardiac myocytes

**DOI:** 10.1085/jgp.20221332908212023c

**Published:** 2023-09-04

**Authors:** Christine Deisl, Jay H. Chung, Donald W. Hilgemann

Vol. 155, No. 10 | https://doi.org/10.1085/jgp.202213329 | August 9, 2023

The authors regret that three errors had been made in the original paper. The corrections are listed here. None of these change the major findings of the article.

The fluorescence profile in Fig. S2 D was a copy of the profile in Fig. S2 C; the correct profile for FITC-albumin at 60 min is shown here. No changes were made to the figure legend. Fluorescence decreases steeply from the pipette tip to the cytoplasm and is approximately constant along the length of the myocyte.

**Figure S2.**
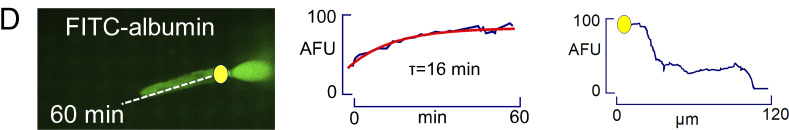


In Fig. 10 F, the x axis value for (Mg)ATP was incorrect in the original article. The corrected panel is shown here; the figure legend remains unchanged.

**Figure 10.**
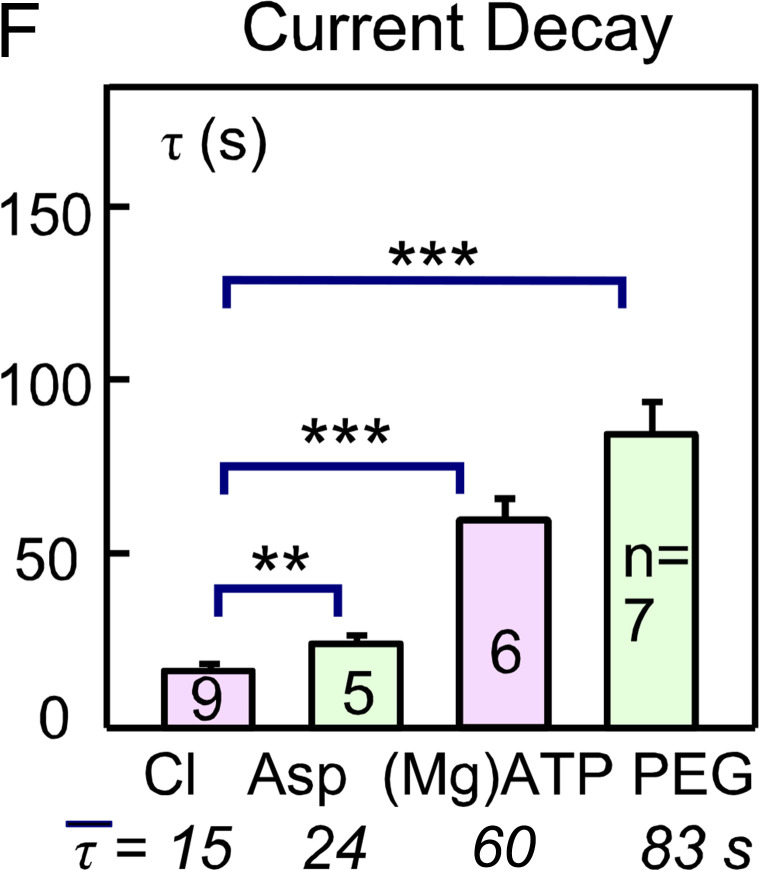


In the penultimate paragraph of the Restricted ATP diffusion in murine myocytes section, the time constants given in the text for the simulations in Fig. 10 G were incorrect. The sentence has been changed to “The time constant for equilibration is 118 s and the time constant of depletion upon activating Na current is 78 s.” These values were correct in the figure.

The errors appear in PDFs downloaded before August 22, 2023.

